# Development of giant cell arteritis after vaccination against SARS-CoV2: A case report and literature review

**DOI:** 10.1097/MD.0000000000033948

**Published:** 2023-06-02

**Authors:** Hiroki Wakabayashi, Misa Iwayanagi, Daiki Sakai, Yoshiya Sugiura, Nobuyuki Hiruta, Yasuo Matsuzawa, Kaichi Kaneko

**Affiliations:** a Division of Respiratory Medicine, Department of Internal Medicine, Toho University Sakura Medical Center, Sakura, Japan; b Division of Rheumatology, Department of Internal Medicine, Toho University Sakura Medical Center, Sakura, Japan; c Department of Surgical Pathology, Toho University Sakura Medical Center, Sakura, Japan.

**Keywords:** adverse reaction, giant cell arteritis, mRNA vaccine, severe acute respiratory syndrome coronavirus 2

## Abstract

Giant cell arteritis (GCA) is an autoimmune vasculitis that affects large and medium-sized blood vessels. The mRNA vaccine against severe acute respiratory syndrome coronavirus 2 (SARS-CoV2) has been associated with the development of immune-mediated diseases. In this article, we present a case of GCA that developed after vaccination against SARS-CoV2.

**Patient concerns:**

A 77-year-old man developed fever, general fatigue, and headache 1 day after the third dose of vaccination against SARS-CoV2. Nodular swelling and tenderness of the bilateral temporal arteries were observed.

**Diagnoses:**

Although right temporal artery biopsies were negative, the patient was diagnosed with GCA based on criteria established by the American College of Rheumatology for the classification of GCA.

**Interventions:**

The patient received methylprednisolone 1000 mg for 3 days. This was followed by prednisolone 1 mg/kg/d, which was decreased by 10 mg every week to 30 mg. From day 16 of hospitalization, the patient received tocilizumab 162 mg/wk every other week.

**Outcomes:**

There was no occurrence of acute side effects. After 38 days of treatment, the condition improved and the patient was discharged from the hospital; as stated above, the dose of prednisolone was tapered to 30 mg/d.

**Lessons:**

We experienced a case of GCA that occurred immediately after vaccination against SARS-CoV2 with an mRNA vaccine. Early signs of GCA include fever, fatigue, and headache, and often resemble those noted after vaccination against SARS-CoV2. The potential presence of GCA should be determined in individuals with persistent fever and headache after vaccination against SARS-CoV2.

## 1. Introduction

Giant cell arteritis (GCA) is an autoimmune vasculitis of large- and medium-sized blood vessels. It typically occurs in patients aged > 50 years and is mainly characterized by headache, fever, and malaise.^[1]^ In 1990, the American College of Rheumatology established criteria for the classification of GCA. According to this classification, the diagnosis of GCA is reached when at least three of the following 5 criteria are met: age ≥ 50 years at disease onset; new onset of localized headache; temporal artery tenderness or decreased temporal artery pulse; elevated erythrocyte sedimentation rate (Westergren) ≥ 50 mm/h; and biopsy sample including an artery, showing necrotizing arteritis characterized by a predominance of mononuclear cell infiltrates or a granulomatous process with multinucleated giant cells.^[1]^ Although genetic, geographic, and hormonal factors have been implicated, the etiology of GCA is unknown.

The relationships between viral infections, vaccine antigens, and the onset or exacerbation of immune-mediated diseases have been thoroughly investigated. Severe acute respiratory syndrome coronavirus 2 (SARS-CoV2) causes a fatal infectious disease, that has resulted in a pandemic. The mRNA vaccine was developed as the most important countermeasure against SARS-CoV2 infection, and has exhibited high efficacy in preventing severe disease. Adverse reactions following vaccination with the mRNA SARS-CoV2 vaccine have been reported. The most common adverse effects are fever (0–21%), headache (0–52.2%), arthralgia (0–35%), and fatigue (8.4–55.4%); rarely, the vaccine is linked to the onset or flare of immune-mediated diseases.^[2,3]^ Several previous reports have described the occurrence of GCA after vaccination against SARS-CoV2.^[4–8]^ Herein, we report a case of GCA that developed after vaccination against SARS-CoV2.

## 2. Case report

A 77-year-old man with type 2 diabetes mellitus, Basedow’s disease, and prostate cancer received 3 doses of an mRNA vaccine against SARS-CoV-2 (BNT162b2; Pfizer–BioNTech, Mainz, Germany). One day later, the patient developed fever, general fatigue, and headache; the symptoms persisted for 3 months. He visited a primary care physician and underwent chest X-ray examination. Following the detection of an abnormality in the chest X-ray examination, the patient was referred to the respiratory department of our hospital.

**Figure 1.**
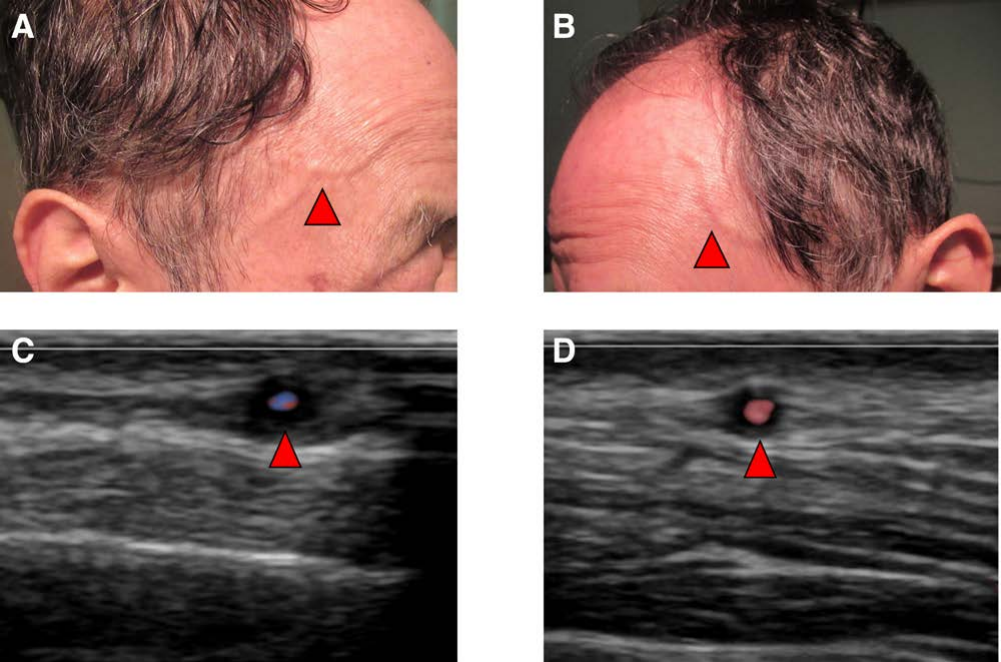
Bilateral temporal arteries of the patient. Nodular swelling of the right temporal artery (A, arrows) and left temporal artery (B, arrows) was observed. Ultrasound echography showing a halo sign surrounding both the right (C, arrows) and left (D, arrows) temporal arteries.

Physical examination revealed a body temperature of 38.0°C, blood pressure of 103/72 mm Hg, and pulse rate of 65 beats/min. Auscultation confirmed that his lungs were clear, abdominal examination was unremarkable without hepatosplenomegaly, and neurological examination yielded normal findings. Nodular swelling and tenderness of the bilateral temporal arteries (Fig. 1A and B), as well as tenderness of the bilateral femoral muscles, were observed. Chest X-ray examination and computed tomography demonstrated slight shadows suggestive of chronic lower respiratory tract infection. Nevertheless, these findings could not explain the cause of symptoms in this patient. Contrast-enhanced computed tomography did not reveal evidence of vasculitis. Laboratory examinations demonstrated elevations in the serum levels of C-reactive protein (13.4 mg/dL), white blood cell count (13.2 × 10^9^/L), and erythrocyte sedimentation rate (62 mm/h). In contrast, the serum levels of creatine kinase, concentration of thyroid hormones, and urinary findings were normal. Antibodies against extractable nuclear antigens, rheumatoid factor, anti-cyclic citrullinated peptide antibodies, and anti-neutrophil cytoplasmic antibodies were not detected. Analysis using polymerase chain reaction for SARS-CoV2 yielded negative results. Further analyses for serum antibodies against the SARS-CoV2 core protein and spike protein were negative and positive, respectively. These results indicated the absence of previous infection with SARS-CoV2.

**Figure 2.**
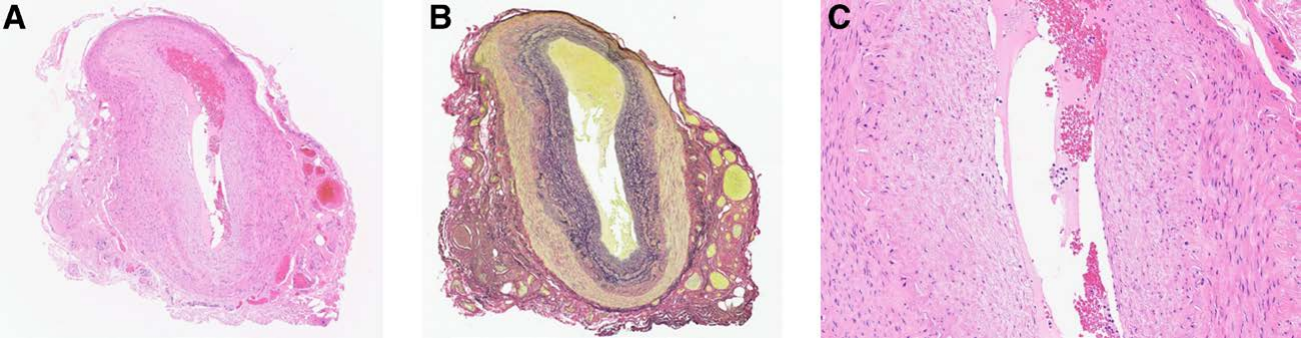
Right temporal artery biopsies. The results did not reveal characteristic findings of GCA, such as inflammatory cell infiltrates or giant cells. Hematoxylin and eosin staining, magnification: ×40 (A); Verhoeff–Van Gieson elastic staining, magnification: ×40 (B); Hematoxylin and eosin staining, magnification: ×100 (C). GCA = giant cell arteritis.

Consequently, GCA was suspected, and the patient was admitted to our hospital for diagnosis and treatment. Ultrasound echography revealed a halo sign surrounding the bilateral temporal arteries (Fig. 1C and D). There was no obvious evidence of vasculitis (i.e., inflammatory cells and giant cells) in right temporal artery tissue collected on day 3 of hospitalization (Fig. 2). According to the criteria established by the American College of Rheumatology, the patient was diagnosed with GCA.^[1]^

**Figure 3.**
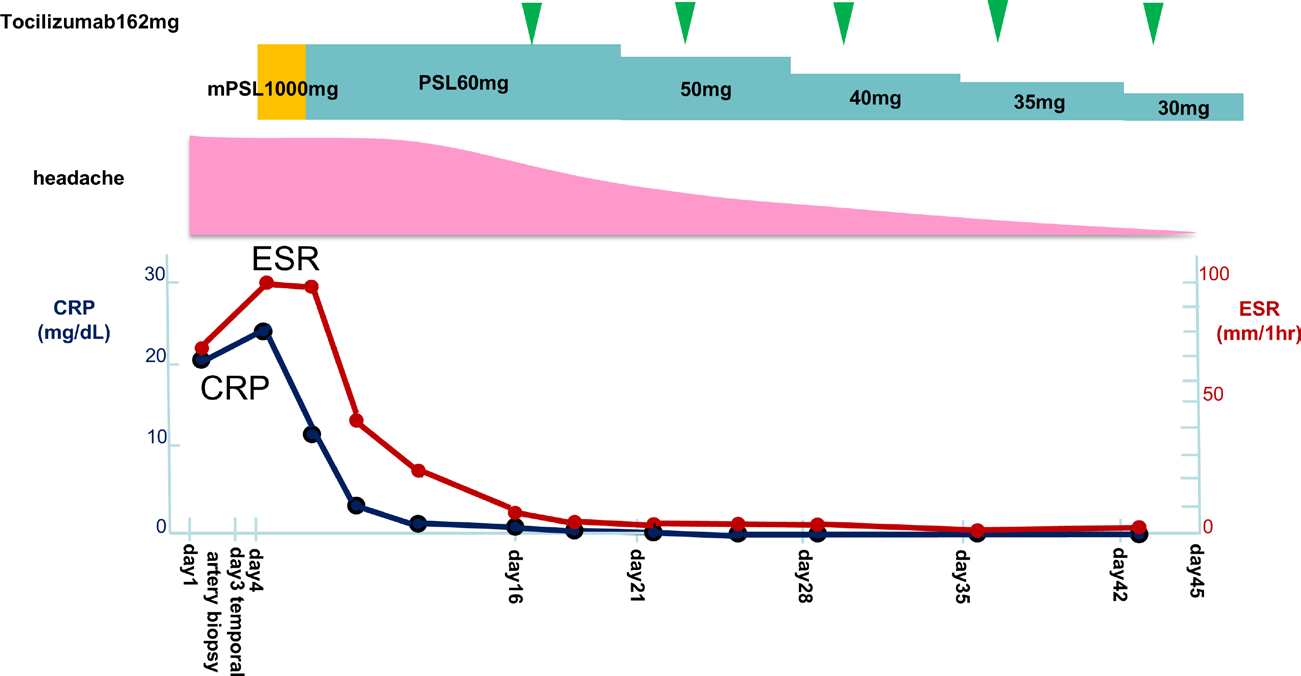
Clinical course of the patient. CRP = C-reactive protein, ESR = erythrocyte sedimentation rate, mPSL = methylprednisolone, PSL = prednisolone.

As shown in Figure 3, he received treatment with methylprednisolone 1000 mg/d on day 4 of hospitalization, and prednisolone 1 mg/kg/d on day 7 of hospitalization. Following treatment, the fever resolved and the headache improved. On day 16 of hospitalization, the patient received tocilizumab 162 mg/wk through subcutaneous injection. There was no recurrence of headache or fever. On day 42 of hospitalization, the dose of prednisolone was tapered to 30 mg/d. Eventually, the patient was discharged from the hospital on day 45.

## 3. Discussion

We reported a case of GCA that occurred immediately after vaccination against SARS-CoV2 with an mRNA vaccine. Several reports have proposed a link between vaccination against SARS-CoV2 with an mRNA vaccine and the development of GCA (Table 1).^[4–8]^ Six patients developed GCA after vaccination against SARS-CoV2; of those, 2 and 4 patients had received the viral vector vaccine and the mRNA vaccine, respectively. The onset time did not depend on the number of vaccinations, and all cases of GCA developed within 10 days after vaccination. The patients were treated with glucocorticoids (all patients), tocilizumab (1 patient), and methotrexate (1 patient), and responded well to the therapy. The present case was similar to previous reports; GCA occurred 1 day after the vaccination, and the patient responded well to treatment. The pathological analysis of our case was negative; however, the diagnosis of GCA was based on the criteria established by the American College of Rheumatology. Previous reports have shown both pathologically positive and negative cases of GCA; 2 of the 6 patients had a positive temporal biopsy, whereas the remaining 4 patients had a negative temporal biopsy or not described. The frequency of positive pathological findings for GCA that developed after vaccination against SARS-CoV2 is lower than that noted for conventional GCA (the sensitivity of temporal artery biopsy in conventional GCA is 92.9%).^[1]^

**Table 1:** Clinical characteristics of 6 cases of giant cell arteritis after vaccination against severe acute respiratory syndrome coronavirus 2.

	Cadiou et al^[6]^	Cadiou et al^[6]^	Anzola et al^[5]^	Greb et al^[4]^	Xia et al^[8]^	Ishizuka et al^[7]^	Present case
Age (yr)/sex	70/female	74/female	83/female	79/male	68/male	74/male	77/male
Number of vaccinations received	1	1	1	2	2	3	3
Time from vaccination to onset of giant cell arteritis	10 d	7 d	1 d	2 d	3–5 d	1 d	1 d
Type of vaccine	Viral vector	mRNA	mRNA	mRNA	Viral vector	mRNA	mRNA
Pathology of temporal artery	Not described	Granuloma, fragmentation of the internal elastic lamina	Normal	Patchy intramural inflammatory infiltrates, multinucleated giant cells	Not described	Not described	Normal
Treatment	Glucocorticoid	Glucocorticoid	Glucocorticoid Methotrexate	Glucocorticoid	GlucocorticoidTocilizumab	Glucocorticoid	Glucocorticoid Tocilizumab
Treatment effectiveness	Effective	Effective	Effective	Effective	Effective	Effective	Effective

The function of mRNA vaccines is based on the following steps: translation of mRNA encoding the viral antigen by intracellular ribosomes; production of the spike protein; presentation of the spike protein as antigen on the cell surface by the major histocompatibility complex; and production of antibodies against the target virus.^[9]^ Studies have investigated the mechanism by which mRNA vaccines induce GCA; the onset of this condition is attributed to cross-reaction of produced antibodies with tissues.^[6]^ In addition, conventional GCA, SARS-CoV2 infection, and adverse reactions after vaccination against SARS-CoV2 promote excessive production of interleukin-6, which may be involved in the pathogenesis.^[6]^ Therefore, the use of an mRNA vaccine against SARS-CoV2 may be linked to the development of GCA.

The present case differs from cases of typical GCA in terms of the negative pathological analysis. Except for pathology, the diagnosis of CGA in previously reported cases that developed after vaccination against SARS-CoV2 was mainly based on a combination of imaging, laboratory testing, and clinical symptom recording. Therefore, GCA that develops after vaccination against SARS-CoV2 may differ from the original one, such as “GCA-like vaccine adverse reaction.” As mentioned above, there are similarities between the adverse reactions associated with mRNA vaccines and symptoms of GCA (e.g., fever, headache, fatigue). Therefore, additional cases should be evaluated to elucidate the pathogenesis of GCA that develops after vaccination against SARS-CoV2.

## Author contributions

**Conceptualization:** Kaichi Kaneko, Hiroki Wakabayashi, Nobuyuki Hiruta, Yasuo Matsuzawa.

**Investigation:** Hiroki Wakabayashi.

**Methodology:** Kaichi Kaneko, Hiroki Wakabayashi, Misa Iwayanagi.

**Validation:** Yoshiya Sugiura, Nobuyuki Hiruta.

**Visualization:** Hiroki Wakabayashi, Misa Iwayanagi.

**Writing – original draft:** Kaichi Kaneko, Hiroki Wakabayashi.

**Writing – review & editing:** Kaichi Kaneko, Hiroki Wakabayashi, Misa Iwayanagi, Daiki Sakai, Yoshiya Sugiura, Nobuyuki Hiruta, Yasuo Matsuzawa.

## References

[1] Hunder GG Bloch DA Michel BA. The American college of rheumatology 1990 criteria for the classification of giant cell arteritis. Arthritis Rheum. 1990;33:1122–8. 2202311 10.1002/art.1780330810

[2] Dotan A Muller S Kanduc D. The SARS-CoV-2 as an instrumental trigger of autoimmunity. Autoimmun Rev. 2021;20:102792102792. 10.1016/j.autrev.2021.102792 PMC7892316 33610751

[3] Sharif N Alzahrani KJ Ahmed SN. Efficacy, immunogenicity and safety of COVID-19 vaccines: a systematic review and meta-analysis. Front Immunol. 2021;12:714170. 34707602 10.3389/fimmu.2021.714170 PMC8542872

[4] Greb CS Aouhab Z Sisbarro D. A case of giant cell arteritis presenting after COVID-19 vaccination: is it just a coincidence? Cureus. 2022;14:e21608. 35228965 10.7759/cureus.21608 PMC8873313

[5] Anzola AM Trives L Martínez-Barrio J. New-onset giant cell arteritis following COVID-19 mRNA (BioNTech/Pfizer) vaccine: a double-edged sword? Clin Rheumatol. 2022;41:1623–5. 35112193 10.1007/s10067-021-06041-7 PMC8810207

[6] Cadiou S Perdriger A Ardois S. SARS-CoV-2, polymyalgia rheumatica and giant cell arteritis: COVID-19 vaccine shot as a trigger? comment on: “can SARS-CoV-2 trigger relapse of polymyalgia rheumatica?” by Manzo *et al*. Joint Bone Spine 2021;88:105150. Joint Bone Spine. 2022;89:105282105282. 10.1016/j.jbspin.2021.105282 PMC8480145 34600148

[7] Ishizuka DK Katayama K Ohira Y. Giant cell arteritis presenting with chronic cough and headache after BNT162b2 mRNA COVID-19 vaccination. QJM. 2022;115:621–2. 35818985 10.1093/qjmed/hcac171 PMC9384497

[8] Xia C Edwards R Omidvar B. A case of giant cell arteritis with a normal erythrocyte sedimentation rate (ESR) post ChAdOx1 nCoV-19 vaccination. Cureus. 2022;14:e25388. 35774715 10.7759/cureus.25388 PMC9236663

[9] Zhang C Maruggi G Shan H. Advances in mRNA vaccines for infectious diseases. Front Immunol. 2019;10:594. 30972078 10.3389/fimmu.2019.00594 PMC6446947

